# SENSITIZATION TO *ASPERGILLUS FUMIGATUS* IN FIBROCYSTICS

**DOI:** 10.1590/1984-0462/;2018;36;3;00018

**Published:** 2018

**Authors:** Nelson A. Rosario, Carlos A. Riedi

**Affiliations:** a Pediatric Respiratory Medicine Service, Hospital de Clínicas Complex, Universidade Federal do Paraná, Curitiba, PR, Brazil.

To The Editor

Cystic fibrosis (CF) and asthma are not always easily distinguished and both can coexist in the same patient.^1^ Reduced pulmonary function, bronchial hyperreactivity, eosinophilia, and elevated IgE levels are common to both.^2^

Specific IgE for at least one aeroallergen, present in 67% by skin tests and 80% by radioallergosorbent test (RAST), indicates that subjects with CF should be evaluated and treated for coexisting allergy.^3^

Patients with CF (n=40, median age: 7.3 years) were investigated for allergic bronchopulmonary aspergillosis (ABPA) by thoracic computed tomography, skin prick/puncture testing (SPT) for aeroallergens, blood eosinophil counts, sputum culture, total serum IgE levels and serum specific IgE to Aspergillus fumigatus (AF) (Immuno CAP^®^).^4^ SPTs were positive in 50% of the patients, and positive in 30% for *D. pteronyssinus*. However, a positive response to AF was observed in 9/40 (23%) and specific IgE ≥0.35 kU/L in 10 (25%) patients. There was a correlation between SPT and specific IgE (p<0.0001). Allergic rhinitis was observed in 22 (55%) patients and 2 had sputum culture positive for *Aspergillus sp*., despite negative SPT/specific IgE being for its antigens. Three patients had a diagnosis of ABPA (Criteria from CF Foundation, USA) with radiological changes (infiltrates and/or bronchiectasis), and of which two of them presented serological ABPA.^4^

Aguiar, Damaceno and Forte found serum specific IgE for *Aspergillus* in 18.6% of their patients (n=86) with CF, as well as positive allergic cutaneous tests (ACT) in 29.5% of them (n=78), results which are similar to those in the present study.^5^ The objective was to know the IgE sensitization index to the AF fungus in cystic fibrosis with serum IgE levels by immunofluoroenzymatic assay and SPT. The SPT technique is not described - probably prick test -, and was considered positive if wheal diameter was ≥3 mm. The presence of allergic diseases and sensitization to other allergens were not evaluated in these patients, which could be a confounding factor in the interpretation of total IgE and SPT results.^5^

Although AF sensitization is crucial for the diagnosis of ABPA in cystic fibrosis, it is necessary to differentiate lung colonization by *Aspergillus*, allergic sensitization and clinically proven ABPA.^2^

Patients with CF should be periodically examined for ABPA, and those sensitized should be monitored more often. Clinical deterioration, high total IgE, and *Aspergillus*-specific IgE are the minimal criteria for ABPA.^4^

Monitoring by SPT and serology, as suggested^5^ and in agreement with what was observed in this study^4^, are useful for the diagnosis of ABPA in fibrocystic patients.
